# RNase A is inhibited by the cysteine-rich protein thionein but not by the metal-containing form metallothionein

**DOI:** 10.1007/s11010-026-05515-z

**Published:** 2026-03-13

**Authors:** Francisco Trujillo-González, Ian Asaf Muñoz-Granados, Brenda L. Sanchez-Gaytan, Eduardo Brambila, Jose Manuel Perez-Aguilar

**Affiliations:** 1 School of Chemical Sciences, Meritorious Autonomous University of Puebla (BUAP), 72570 University City, Puebla Mexico; 2 Chemistry Center, Science Institute, Meritorious Autonomous University of Puebla (BUAP), 72570 University City, Puebla Mexico

**Keywords:** Thionein, RNase A, Protein-protein interaction, Proinflammatory stress, Protein synthesis, Molecular dynamics (MD) simulations

## Abstract

Metallothionein (MT) is a cysteine-rich protein that displays a high redox activity, acts like an acute phase protein, and forms metal-thiolate clusters. However, little is known about how the presence of metals in MT or their absence in Thionein (T) modify the structure of the protein and if such differential conformations alter the protein-protein interactions that MT/T establishes with other proteins. Ribonucleases (RNases) are a group of enzymes that hydrolyze different classes of RNA and due to the relevance of their function, they are highly regulated by specific inhibitors. Earlier results showed a decrease in RNase activity during an inflammatory process that appears to be a consequence of a *de novo* synthesis of an unidentified RNase inhibitor (RI) (Brambila et al in Exp Mol Pathol 71:125–131, 10.1006/exmp.2001.2383 , 2001). Therefore, we performed experimental studies to evaluate the ability of MT/T to inhibit RNase A activity in vitro. Computational methods were utilized to characterize the tertiary structure of MT/T in the presence and absence of zinc ions and to evaluate the stability of a complex with RNase A. Our results show that only in the absence of metals, T, the homodimeric protein has an inhibitory effect on RNase A activity via a mechanism that involves the formation of a complex between RNase A and T by specific interactions. Also, the involvement of residues, including cysteines that coordinate the cations, that participate in intermolecular interactions in the metal-bound form, aids in explaining the metal dependent inhibitory properties in the metal-free protein. This work suggests an additional role of T in the regulation of enzyme activity and protein synthesis.

**Supplementary Information:**

The online version contains supplementary material available at 10.1007/s11010-026-05515-z.

## Introduction

The study of the structure, function, and interactions of proteins is essential to have a complete understanding of the different signaling pathways that take place inside cells. In this context, the formation of protein-protein complexes is central for various cellular processes including cell recognition, signal transduction, immune response, assembly of cellular components, and regulation of enzymes’ activity [1, 2]. The interactions between proteins are essential to maintain a homeostatic state and many human diseases can be traced to aberrant protein–protein interactions, by either the formation of a protein complex at an inappropriate time and/or location or the loss of an essential interaction [3].

Thus, there is a growing interest in understanding how the three-dimensional structure of a protein relates to its function and how structural changes affect the ability of proteins to interact and selectively modulate other proteins.

Metallothionein (MT) is a small cytosolic protein (between 61 and 68 residues) that exhibits a high content of cysteine residues with a high redox activity. The 20 conserved cysteine residues present in the structure of MT form metal-thiolate clusters, which can coordinate up to seven metals in its structure, including zinc, cadmium, copper, and mercury [4, 5].

In mammals there are four isoforms, namely, MT-1, MT-2, MT-3, and MT-4. The two predominant isoforms, MT-1 and MT-2, are ubiquitous in all organs while MT3 and MT4 are mainly expressed in the central nervous system and specialized tissue (e.g., the stratified epithelium), respectively [4, 6, 7]. The functions associated to MT proteins are various including homeostasis, regulation and metabolism of metals, participation in cellular stress and inflammation processes (acting as an acute phase protein), regulation of neurogenesis, neurodegeneration, and tumorigenesis processes [4–9].

Under normal physiological conditions, MT forms dimers that exist in a dynamic equilibrium between three redox states: an oxidized form (namely, thionin), a reduced form (namely, thionein), and a metal-bound form (namely metallothionein), with the last two as the most relevant species [10–13].

The first structural investigations focused mainly on the structural properties of the metal-coordinated holoprotein MT while details regarding the metal-free protein Thionein (T) remain largely unknown [14].

**Fig. 1:**
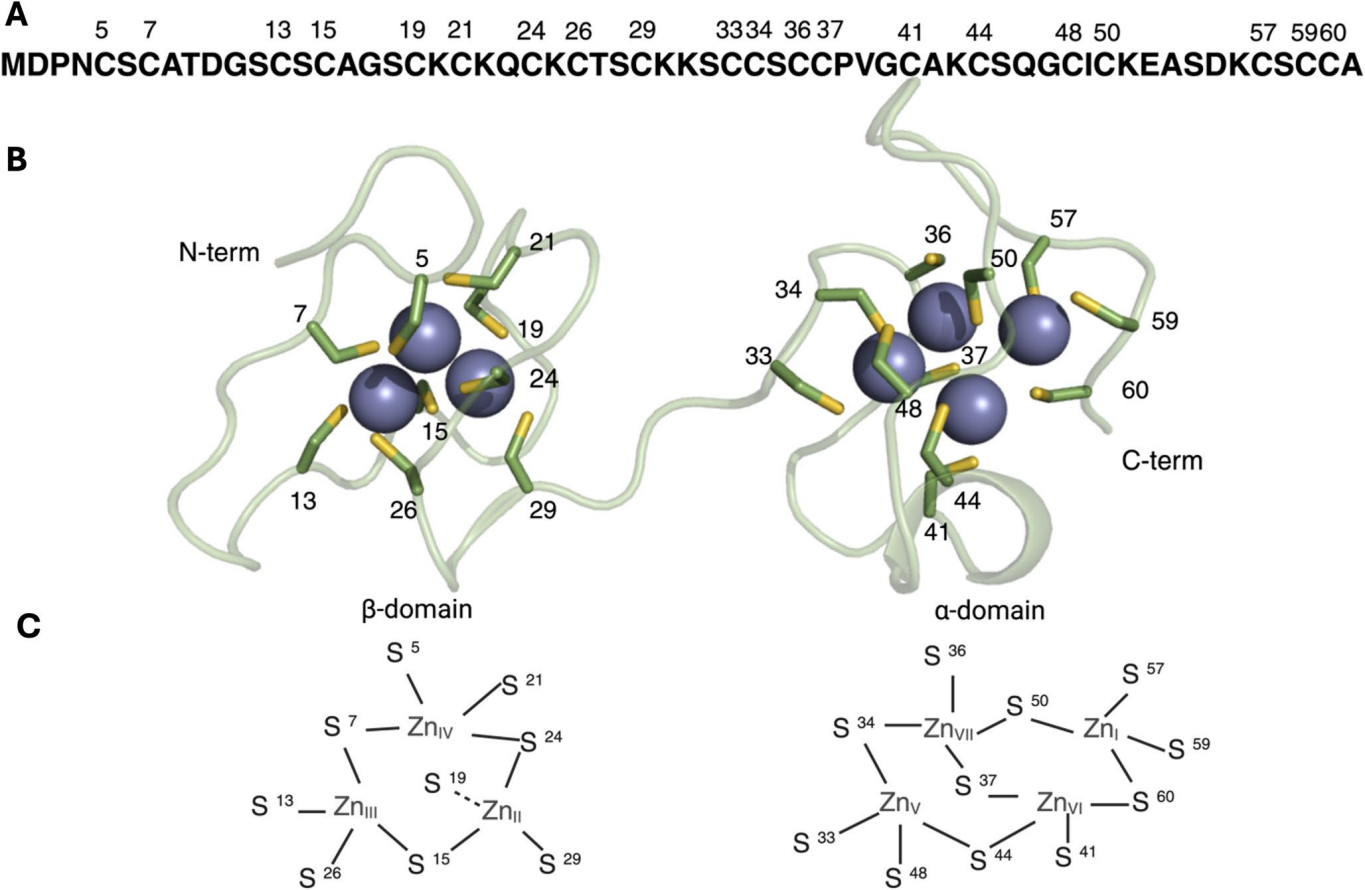
Structure of MT2. **A** Sequence of MT2 where the residue number of all the cysteine residues is represented on top of the sequence. **B** Structure of the Zn_7_-MT2 complex displaying three cations in the β-domain and four in the α-domain. Zn(II) are represented as spheres in grey whereas the Cys residues (in their thiolate form) are depicted as sticks. Interestingly, there are neither α-helical nor β-strand segments in the experimental structure of the Metal_7_-MT2 complex **C** Schemes of the Zn(II)-thiolate clusters present in the β- and α-domains of MT

The 3D structure of MT contains several half-turns and two helical segments and presents two domains, a N-terminal domain denominated β-domain and a C-terminal domain denominated α-domain, The β-domain contains 9 cysteine residues forming a metal-thiolate cluster able to coordinate up to 3 metal ions (M_3_Cys_9_) whereas the α-domain contains 11 cysteine residues forming a metal-thiolate cluster able to coordinate up to 4 metal ions (M_4_Cys_11_); with every bound metal ion presenting a tetrahedral coordination environment (Fig. 1) [10, 15, 16].

Although there is available information regarding the three-dimensional structure of the metal-bound form MT obtained by both X-ray crystallography and NMR [17–21], details regarding the structural stability in the absence of metal remain unknown.

While the metalated form MT is predominant under physiological conditions, and a dynamic pool of partially metalated species has been described (with Zn_5_MT and Zn_6_MT being the dominant forms), under certain pathophysiological contexts the expression of the metal–free form, T, becomes significantly elevated [12, 22–24]. Even though T is typically metalated shortly after synthesis under physiological conditions (transitioning to MT), emerging evidence suggests that T may acquire functional relevance during acute inflammatory process, metal imbalance, and oxidative stress responses. In such contexts, de novo synthesis of T as an acute-phase protein may exceed the immediate availability of metal ions, resulting in a transient pool of bioavailable T. This form has been shown to act as a redox-sensitive buffer, capable of scavenging reactive oxygen species (ROS) through oxidation of its cysteine residues and dynamically modulating intracellular zinc levels [24, 25]. Moreover, T may participate in signaling pathways related to immune activation and apoptosis, particularly in tissues exposed to lipopolysaccharide (LPS), TNF-α, or IL-6 [23, 24, 26].

MT can interact with various proteins; in the mammalian kidney, MT1/MT2 interact with megalin and the transthyretin transporter, brain MT3 seems to control the loading of metal ions on peptides whose aggregation leads to neurodegenerative disorders, such as the Aβ peptide, α-synuclein, and prion proteins. The interaction of MTs with zinc-dependent enzymes and transcription factors (such as p53, NF-kB and PKCµ) can activate/deactivate them, thus conferring on MTs the role of metabolic regulators and gene expression. Interactions of MT with ferritin and bovine serum albumin have also been documented [5, 27, 28].

It is known that T can regulate indirectly the activity of transcription factors such as TFIIIA, Zn-Sp1, and p53 by competing for zinc bioavailability, however, studies have shown that T can act directly and selectively given that T can inhibit p53 and only metal-free MT1, and not MT1, forms a complex with p53 [29].

Therefore, it is of special interest to investigate how the presence or absence of metal contributes to modulating the different conformations of MT/T and if such conformations modify the interplay of MT/T with the function of other proteins.

Ribonucleases (RNases) are a wide group of enzymes that hydrolyze different types of RNAs via 2´, 3´-cyclicCMP to form oligo- or mononucleotides with a terminal 3´-phosphate intermediate [30–32]. Ribonucleases can be classified by the type of RNA they hydrolyze, by their structure, by the optimal pH at which they function, or even a classification that differentiates intracellular ribonucleases from those secreted into extracellular fluids, called secretory ribonucleases [31, 33].

Among the alkaline (optimal pH between 7 and 8) and secretory ribonucleases is the bovine pancreatic ribonuclease type A, a 124-residue protein, considered the most studied ribonuclease of the 20th and 21 st centuries. Subsequently, the identification of ribonucleases whose sequence and structure are highly similar to bovine pancreatic ribonuclease, constituted the family of type A ribonucleases [33–35].

**Fig. 2:**
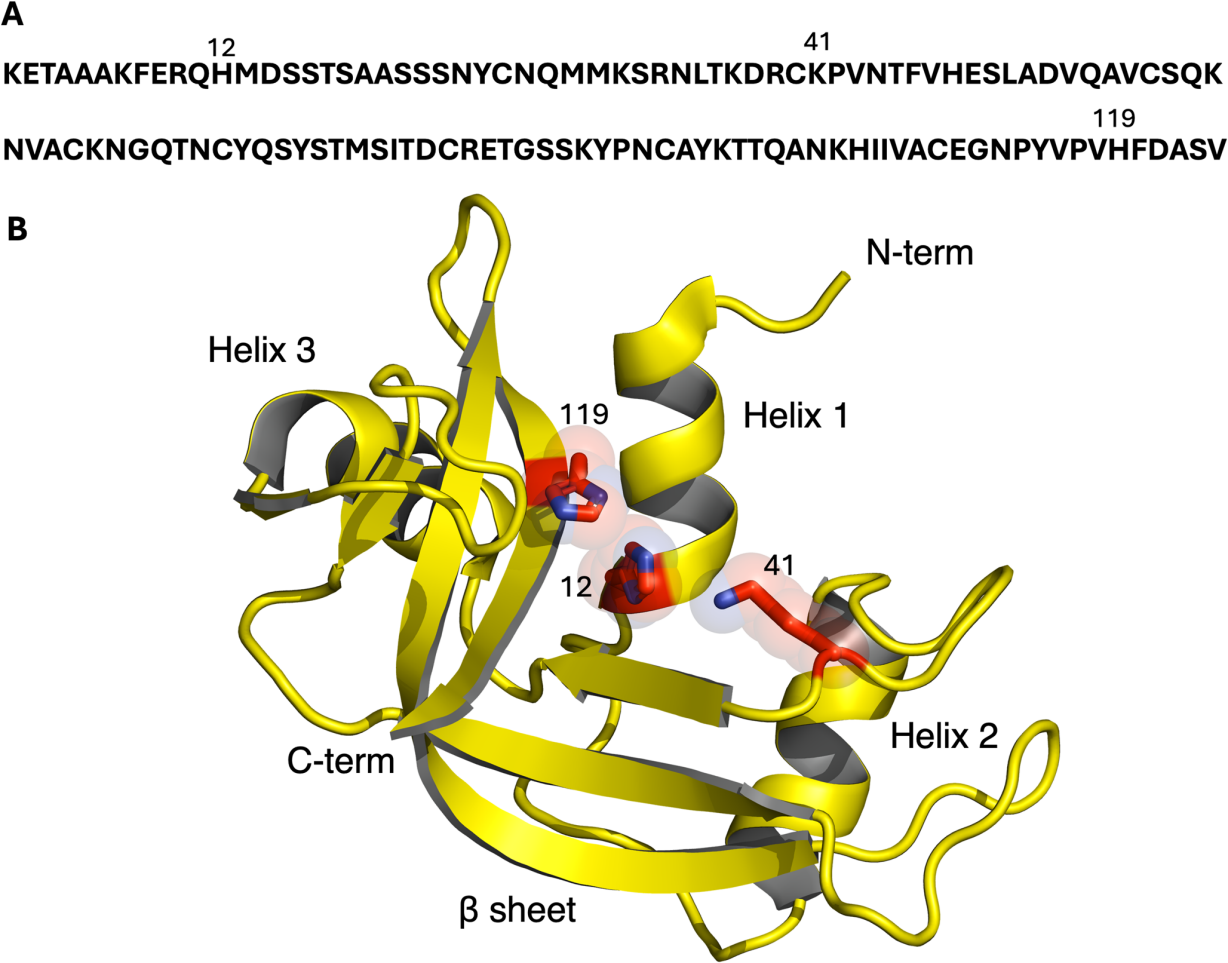
Structure of RNase A. **A** Sequence of RNase A where the residue number of the catalytic residues, H12, K41, and H119, is indicated on top of the sequence. **B** 3D-structure of monomeric RNase A. The catalytic triad (H12, K41 and H119) are represented as red sticks. The three helices and the β bridge of the structure are represented as cartoons

The three-dimensional structure of ribonuclease A exhibits a kidney shape consisting of a large twisted β-sheets stabilized by four disulfide bridges that present a short helix at the N-terminus along with two additional short single-turn helices positioned elsewhere in the structure of the protein. The active site resides in a pronounced cleft in the center of the kidney structure, and includes residue Lys41, whose amino group binds the target phosphate group of the RNA substrate, residue Thr45, which selectively binds pyrimidines, and two histidine residues, His12 and His119, which are responsible for catalyzing the hydrolysis of the phosphodiester bond (Fig. 2) [36].

Ribonuclease A possesses a physiological inhibitor (RI) with a high affinity (K_d_ in the picomolar to femtomolar range) that strongly inhibits its function. Nonetheless, ribonuclease A can still maintain its activity by phosphorylation at some Ser/Thr residues that are crucial for RI binding or by oligomerization forming various types of catalytically active oligomers through a domain swapping mechanism [37–41].

Previous studies showed that decreased RNase activity following surgical trauma appears to be a consequence of an inhibition mechanism by a “*de novo*” protein synthesis of an unidentified RNase inhibitor [1].

While Ribonuclease Inhibitor (RI) is constitutively expressed across various tissues without evidence of inflammation‑induced *de novo* synthesis [37, 42, 43], Metallothionein (MT) functions as an acute‑phase protein. MT synthesis is upregulated *de novo* during surgical and non-infectious inflammatory conditions, facilitating the redistribution of zinc from systemic circulation to hepatic intracellular compartments [26, 44].

Given that metal binding modulates the inhibitory capacity of this novel inhibitor [1], the high metal content and inducible nature of MT suggest it is a compelling candidate for evaluating RNase A inhibition within inflammatory and postoperative contexts. Furthermore, previous reports indicate that the metal-free apo-form (T) selectively inhibits p53 [29], suggesting that MT and T possess distinct inhibitory profiles. Consequently, a comparative assessment of both MT and T is essential to delineate which conformational states confers RNase A inhibition under specific physiological or pathological conditions.

Prompted by this evidence and using a combination of experimental and computational approaches, we investigated if the presence or absence of metals in the structure of MT/T affects the protein’s capacity to interact with RNase A. Our results indicated that only in the absence of metals, i.e., zinc, the interaction of T with RNase A is observed. Moreover, the formation of the T/RNase A protein complex inhibits the function of ribonuclease providing a novel metal-dependent regulatory mechanism for this relevant enzyme.

## Materials and methods

All materials used were treated for 24 h in nitric acid diluted in deionized water (1:19 v/v) and thoroughly rinsed with distilled and deionized water to prevent possible metal contamination.

The solutions used in the determinations, as well as the buffer solutions, were prepared using deionized water.

### Animals and treatment

Wistar rats (3) weighing 240–250 g were administered i.p. two times with an interval of 24 h with 1 mg/Kg of CdCl₂ to induce MT synthesis. Then, livers were excised from the rats previously anesthetized with xylazine/ketamine (20/137 mg/Kg) and perfused with NaCl (0.9%).

### Isolation and purification of MT

Rat livers were homogenized in Tris-Cl 50 mM, pH 8.0, NaN_3_ 0.02% and centrifuged at 3000 g for 20 min at 4 °C. Supernatants were subjected to a heat treatment to eliminate thermolabile proteins and centrifuged at 3000 g for 20 min at 4 °C. MT in the supernatant was isolated by molecular weight exclusion chromatography using Sephadex G-75 and Tris-HCl 10 mM, pH 8.0, NaN_3_ 0.02% (Flow rate 0.6 mL/min, and 6.0 mL fractions were collected). The presence of MT in each fraction was monitored by UV absorption at 254 nm, and MT-Cd content was identified by atomic absorption (Figure S1). MT fractions were concentrated with an Amicon system and re-chromatographed to ensure MT purification. MT was stored at − 80 °C. MT isoforms were isolated using ionic-exchange chromatography on a DEAE-Sephadex A25 column. Elution was performed with a linear concentration gradient of Tris-acetate (2–250 mM), and 5 mL fractions were collected. MT-1 and MT-2 isoforms were identified spectrophotometrically by monitoring absorbance at 250 and 280 nm.

### T preparation

Isolated MT was incubated in the presence of dithiothreitol for 24 h and acid treatment at pH 1.0 to favor the release of the metal ions and to obtain T. To eliminate the released metal ions and concentrate the T, this solution was filtered using centrifugal filters with YM-3 Ultracel membranes (Microcon). Then T was quantified and characterized by UV-Vis spectroscopy at 220 and 254 nm and stored at − 80 °C.

RNase A from bovine pancreas was obtained from a commercial source (Sigma, St Louis, MO).

### Inhibition of RNase A activity by MT and T

The RNase A activity was determined by measurement of 2´, 3´-cCMP in Tris/acetate 0.5 M, pH 6.5, 5 mM EDTA at 25 °C. Enzyme activity was quantified by the increase in absorption at 286 nm. One enzymatic unit was defined as an absorbance change of 0.0146 at 286 nm [45].

To determine the inhibitory effect of metallothionein (MT) and thionein (T) on RNase A enzymatic activity, 1.0 mg of the enzyme was mixed in the reaction buffer with increasing concentrations of MT or T (0–1.6 nM). Following mixing, residual enzymatic activity was quantified. To evaluate the potential recovery of RNase A activity, ZnCl_2_ (0–6 nM) was added to solutions containing the enzyme previously inhibited by T. The mixture was incubated for 3 and 10 min to assess the restoration of RNase A enzymatic activity.

### SDS-PAGE electrophoresis

The RNase A/T complex was identified by sodium dodecyl sulfate-polyacrylamide gel electrophoresis (SDS-PAGE). Samples of T, RNase A, a mixture of RNase A/T, and RNase A/T/ZnCI_2_ were placed in separate wells of 15% Tris-Tricine gel, and electrophoresis started under standard conditions. Gel was stained with Coomassie Blue R-250 for 60 min.

### System preparations for MD simulations

The MT structure was obtained from the PDB ID **4MT2** [46], which corresponds to a rat (r*attus rattus*) dimeric MT-2 crystallographic structure. This structure was resolved with 5 Cd(II) and 2 Zn(II) ions. For this structure of MT, we replaced the 5 Cd(II) ions with 5 Zn(II) ions in both chains to replicate the experimental conditions of the Zn-saturated form of MT. For the metal-free T dimeric initial configuration, the same PDB structure was utilized, but with all the metal atoms removed. The sidechain of the cysteine residues in T was treated as thiols (reduced form), while the MT cysteine residues were treated as thiolates based on the MT/T redox cycle [11–13, 16]. The protonation states for the remaining amino acid residues were those more likely to be present at a neutral pH. Both systems were solvated and ionized with a TIP3 water model using a 0.15 M salt concentration of NaCl. The biomolecular systems were prepared using the VMD software [47].

The RNase A structure was obtained from the PDB ID **1FS3** [48], which corresponds to a bovine (*Bos taurus*) crystallographic structure.

The heterotrimeric complex of dimeric T and monomeric RNase A was prepared under the same physiological conditions as the MT and the T systems.

### All-atom MD simulations

To obtain a representative structure under physiological conditions of temperature, pressure, and salt concentration for the metal-free protein T and the metal-bound protein MT, all-atom molecular dynamics simulations were carried out. MD simulations of the two systems were conducted for 500 ns using NAMD [49] and the all-atom CHARMM36 force field [50, 51]. The conditions used to simulate a physiological environment in the systems were 310.15 K of temperature and 1 atm of pressure. All unbiased MD simulations were performed with a 2.0 fs time step.

To evaluate the stability of the T-RNase A protein complex and the residues involved in the protein-protein interactions, unbiased all-atom MD simulations were conducted for 1.0 µs using the same conditions as in the dimeric MT and T systems.

### Protein-protein docking

A representative structure of T was taken from the trajectory at 300 ns of simulation (based on structural stability). To obtain a T-RNase A interaction mode, a consensus protein-protein molecular docking scheme was performed. HADDOCK [52, 53], Cluspro [54, 55], HawkDock [56] and AlphaFold Multimer [57, 58] software and webservers were used in the consensus docking scheme. The best pose obtained was correlated with known structural experimental information of RNase A with RI (PDB IDs: **1Z7X** [59] and **1DFJ** [60]).

### Interaction energy analysis

To evaluate the interaction energy of the T-RNase A complex, we used the NAMD energy tool [49], using a 12-cutoff value for nonbond interactions and a 10 value for switching distance. We measured and characterized electrostatic energy, van der Waals energy, and total energy of the complex.

## Results and discussion

### Enzymatic evaluation of the activity of RNase A with MT/T

**Fig. 3:**
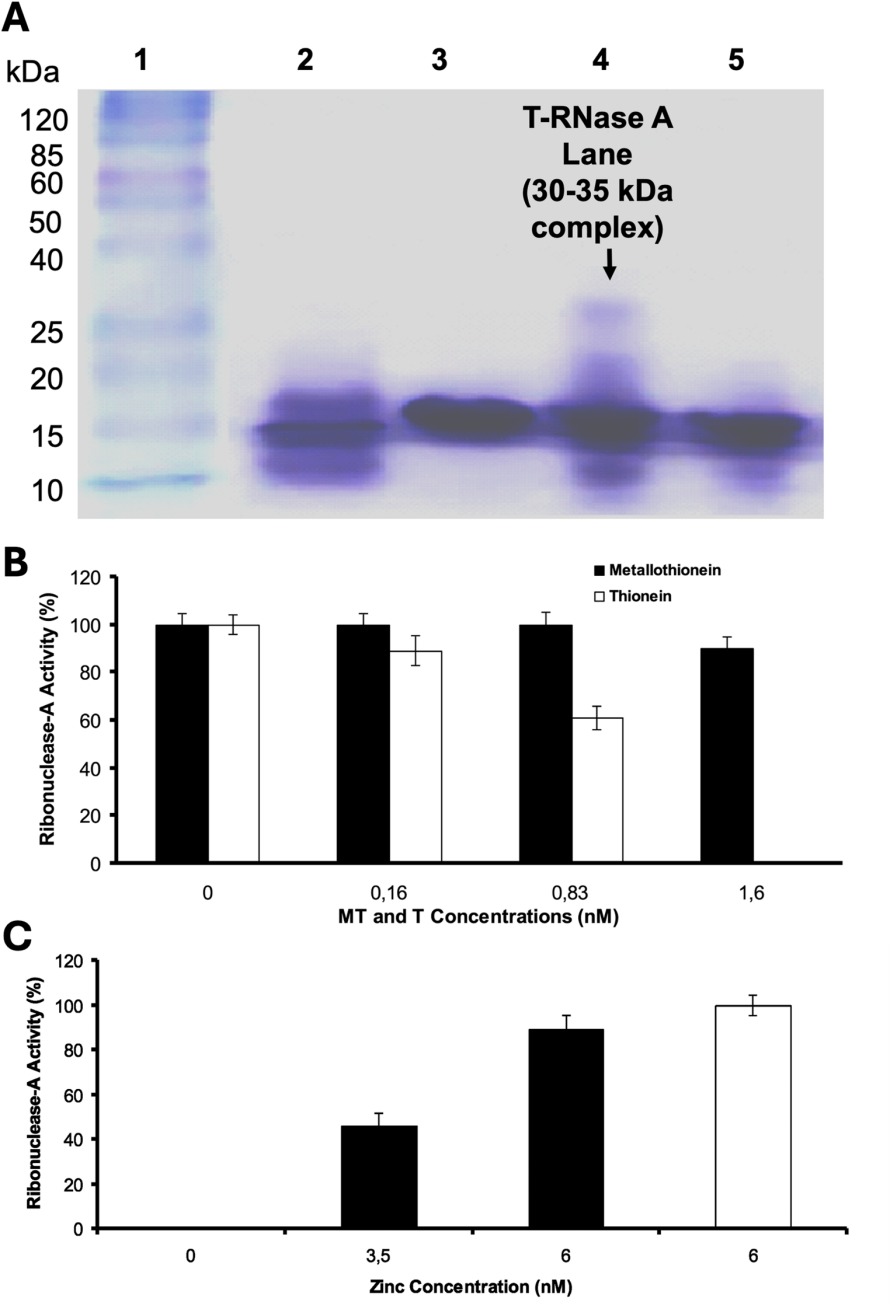
**A** SDS/Page Tris Tricine Electrophoresis. Lane 1: Molecular weight standards, Lane 2: Thionein, Lane 3: RNase A, Lane 4: Thionein and RNase A, Lane 5: Thionein, RNase A and Zn(II). A 30–35 kDa complex is observed in Lane 4. **B** Ribonuclease inhibition by MT and T determined by the 2´, 3´-cCMP hydrolysis after the measurement of 3´CMP at 286 nm. Only T shows a significant inhibitory effect on RNase activity. Data represent means (*N* = 3). RNase A enzymatic activity was determined at pH 6.5 and 25 °C, conditions under which the enzyme exhibits maximal catalytic activity. While RNase A remains active under physiological conditions, it does to a lesser degree. **C** RNase A activity restoration after Zn(II) addition. Zn(II) addition to the solution containing completely inactivated RNase A by T restores enzymatic activity. Maximum RNase reactivation (89%) was obtained after the addition of 6 nM of Zn(II) to the solution. RNase reaches 100% reactivation after RNase-T incubation at 37 °C by 10 min. Data represent means (*n* = 3)

The RNase activity measured by the formation of the product 3´CMP in the presence of MT/T on an enzymatic assay showed that only T has a significantly inhibitory effect on the RNase A activity (Fig. 3B). The addition of Zn(II) to the solution containing partially inactivated RNase A (61% activity) or completely inactivated RNase A restored the enzymatic activity (Figure S3). Maximum RNase A reactivation (89%) was obtained after the addition of 6 nM of Zn(II) to the solution. RNase A reached 100% reactivation after RNase-T incubation at 37 °C for 10 min (Fig. 3C). Results obtained also showed that the apo-MT-1 isoform has the same inhibitory effect as total T (Figure S2). These experimental results showed that MT does not exhibit an inhibitory effect on RNase A activity; however, T inhibits RNase A activity in a concentration-dependent manner.

### SDS/PAGE electrophoresis results

To investigate the direct physical interaction between thionein (T) and RNase A, we performed Tris-Tricine SDS-PAGE (electrophoresis) to identify the putative T-RNase A complex formation. As shown in Fig. 3A, the first lane contains molecular weight markers (10–120 kDa), while the second and third lanes contain RNase A and T controls, exhibiting bands at approximately 13.7 kDa and 14 kDa, respectively. In the fourth lane, incubations of T and RNase A resulted in the appearance of a band between 30 and 35 kDa, corresponding to the T-RNase A. Given that MT monomers typically range from 7 to 10 kDa and that the biological unit is the dimeric form (14–20 kDa) [61, 62], the observed molecular weight of the complex is consistent with an assembly involving dimeric T and monomeric RNase A. Furthermore, the addition of a seven-fold molar excess of zinc (relative to T) led to the disappearance of the T-RNase A complex (lane 5). This suggests that the introduction of zinc promotes the formation of metal-bound MT, which lacks inhibitory effects on RNase A, correlating with our previous enzymatic assays.

### Computational modeling of the MT/T conformations

Based on our experimental results, we used computational techniques to investigate the structural basis of the metal-dependent inhibition of the function of RNase A by the metal-free T protein. First, both the homodimeric MT and T systems were prepared as indicated in the Materials and Methods section. Briefly, the structure of the mouse MT was obtained from the PDB (accession code 4MT2). The structure consists of two monomers (herein chain A and chain B) each coordinating 7 divalent cations (5 Cd(II) and 2 Zn(II)). To recreate the experimental conditions of the Zn-saturated form of MT, Zn(II) was used in the 7 cation locations (homodimeric Metal_7_-MT2). The coordinated cysteine residues bear the thiolate group (deprotonated form). In the case of the metal-free form of the protein, T, the same structure was used but with all the cations removed, and the cysteine residues were modified as thiols (reduced form). Both systems were solvated and investigated by all-atom MD simulations under physiological conditions of pressure and temperature (see Materials and Methods); both systems, MT and T, were investigated by 500-ns-long MD simulations.

To examine the structural stability and global conformational changes that the MT and T proteins can adopt, we performed Root Mean Square Deviation (RMSD) and secondary structure analyses.

In the RMSD analysis, we identified that both dimeric systems—MT and T—reached a structural stabilization at approximately 150 ns for both chains. As expected, the metal-bound MT structure shows lower structural divergence, relative to the initial conformation.

In the case of the MT system, the RMSD analysis of each of the MT chains showed a similar pattern of structural stability reaching a plateau with RMSD values below 10 Å, relative to the initial structure, for both chains. Interesting, and in comparison to the experimentally solved 4MT2 crystallographic structure, in aqueous media the final structure of the MT systems exhibit a rotation of approximately 80° of one monomer relative to the orientation of the other, which causes that the overall RMSD value of both chains to be slightly higher than that of the individual chains; nevertheless, each monomer stably retains both its secondary and tertiary structural content as well as the structure around the metal coordinating sites (see Figure S4).

**Fig. 4:**
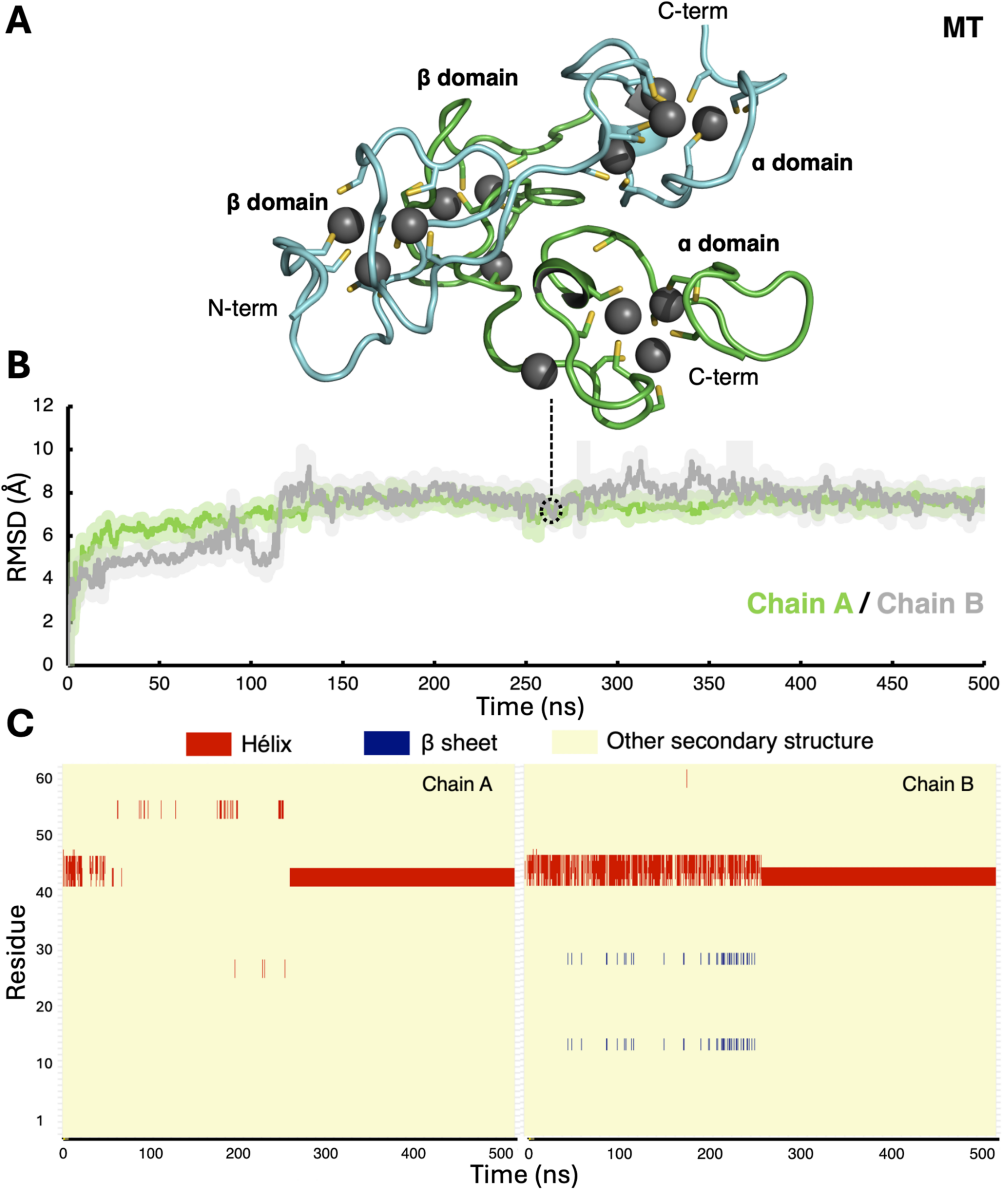
**A** Structural changes of the MT structure. The structure of homodimeric MT at 260 ns of the MD simulation is shown with chain A colored green, chain B colored cyan, and the Zn(II) ions depicted as gray spheres. **B** RMSD analysis of chain A and chain B of the dimeric MT structure. Chain A is shown in green and chain B in gray. **C** Secondary structure analysis of all the residues of MT, where chain A is shown on the left panel and chain B on the right panel. The secondary structures were assigned by the STRIDE program as installed in VMD [47]. Helical content is presented in red, β sheets in blue, and other secondary structures (like coils and turns) in yellow

The available structure of the homodimeric Metal_7_-MT2 complex does not exhibit β-strands and only a short α-helical segment is present around residues 42 to 45. The secondary structure analysis of MT revealed that segments with defined secondary structure in the initial system remain stable during the time of simulation. In chain A, the helical segment in the α domain fluctuated between helical and unstructured conformations around the first half of the MD trajectory (~ 0 to 240 ns), before achieving stable helical conformations for the rest of the simulation (240 to 500 ns). Similar behavior was observed in the case of chain B although in this case, no loss of the helical content was observed (Fig. 4). Notably, during the entire MD simulation, all the Zn(II) ions remained coordinated in the homodimeric structure of MT, highlighting the importance of metal ions in the structural stability of the metal-containing protein, which is correctly captured by the computational technique.

**Fig. 5:**
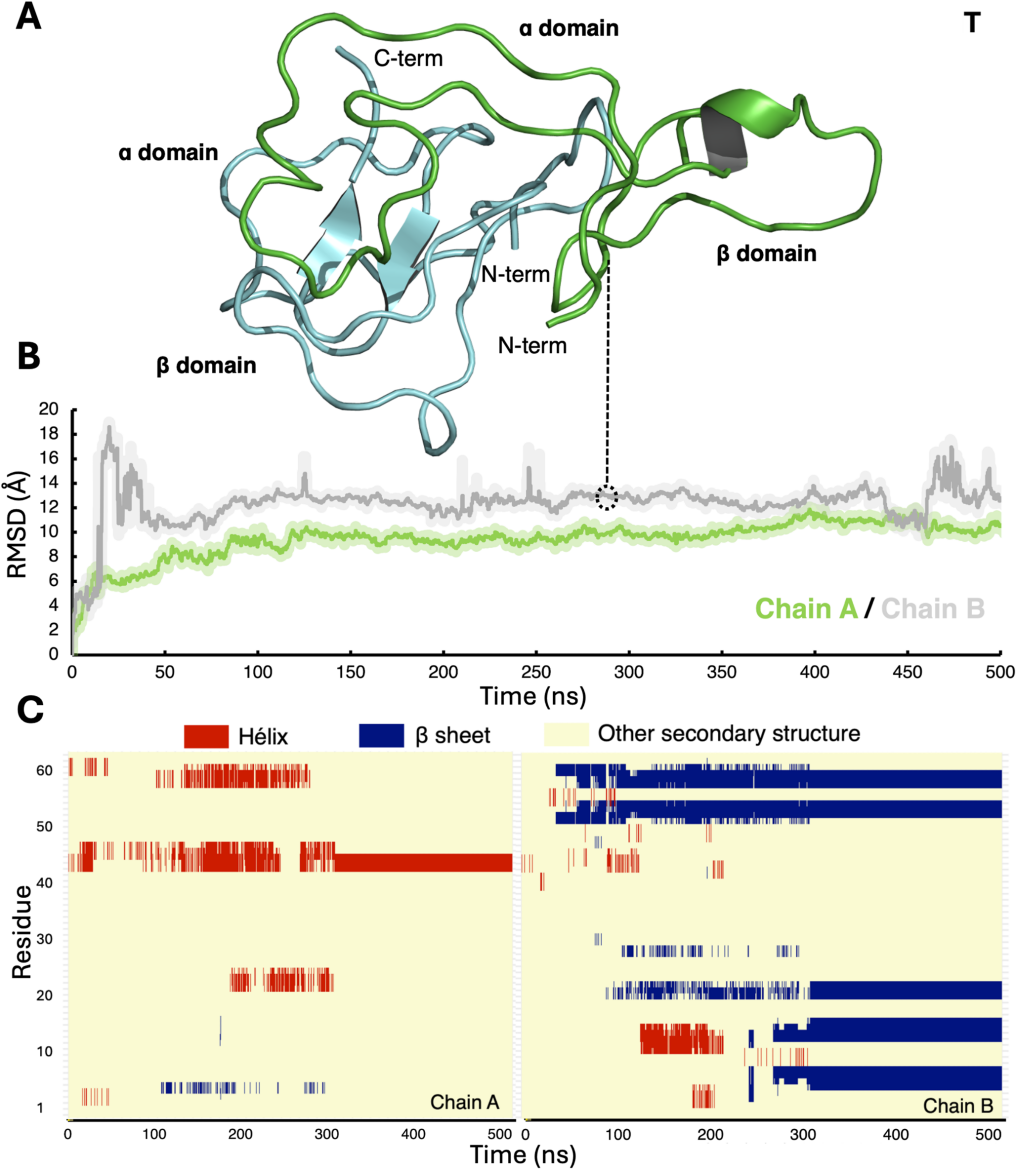
**A** T structure at 300 ns of MD simulation. Chain A is represented as cartoon in green, chain B as cartoon in cyan. **B** RMSD analysis of chain A and chain B of the dimeric T structure. Chain A is shown in green and chain B in cyan. **C** Secondary structure analysis of all the residues of T, where chain A is shown on the left panel and chain B on the right panel. The secondary structures were identified by the STRIDE plugin in VMD [47]. Helices are represented in red, β sheets in blue and other secondary structures (like coils and turns) in yellow

In the case of the metal-free T system, the RMSD analysis identified that, relative to the initial structure, both chains A and B, display larger values than the metal-bound system. Particularly, chain B explores significant conformational changes with RMSD values higher than 10 Å; chain A shows a more stable global conformational changes comparable with thus in the metal-bound chains. The secondary structure analysis of the T system exhibited a significant number of changes in the structure such as the formation of stables β sheets in both the α and β domains of chain B, the most dynamic of the chains based on the RMSD values (Fig. 5). Also, helical structures are partially formed in the first half of the simulation in the β domain of chain B. As for chain A, a helical segment is formed around residues 42 to 45, similarly as that observed in the metal-bound system, MT.

Hence, we identified that T, in the absence of divalent cations, could adopt stable conformations in a physiological context, albeit more structurally diverse, that are different from the known structure of the metal-containing form, MT. These results are consistent with previous computational analyses carried out on the demetallation of Cd-bound MT-1 that suggested that the metal free protein is structurally stable as well as with FRET and ESI-MS analysis, where it was found that that T retains a compact fold upon demetallation at neutral pH and the overall dimensions of the protein did not change significantly from the metal-bound to the metal-free states [14, 22, 61–66].

### Interaction analysis of the T-RNase A complex

The three-dimensional structure of RI-RNase A complex was determined in 1995 and the three-dimensional structure of the human ribonuclease (Ribonuclease 1) with RI was determined in 1997. From the obtained structure of the RI-RNase 1 complex it was possible to identify residues (e.g., Arg39 and Arg91) that are responsible for its remarkable stability [59, 60, 67]. To identify the best pose of the T-RNase A complex, we correlated the best pose obtained by the consensus protein-protein docking scheme (Figure S5) with the previous structural and experimental known information regarding the RI in complex with RNase A. We identified that T is positioned in a similar region to that used by RI, where interactions with residues Arg39 and Lys91 (Arg91 of RNase 1 is replaced by a Lys91 in RNase A, maintaining its characteristics as a positively-charged amino acid residue), knowing to be fundamental residues for the stability of the complex with RI, are observed (see Figure S6). Also, we identified the presence of interactions between T and RNase A catalytic residues Lys41, Thr45, His12, and His119 [59].

To elucidate the stability of the proposed T-RNase A complex, 1.0 µs-long MD simulations were performed under physiological conditions.

We evaluate the protein complex stability as well as interacting determinants during the entire unbiased MD simulation based on a series of structural and protein-protein interaction analyses.

**Fig. 6:**
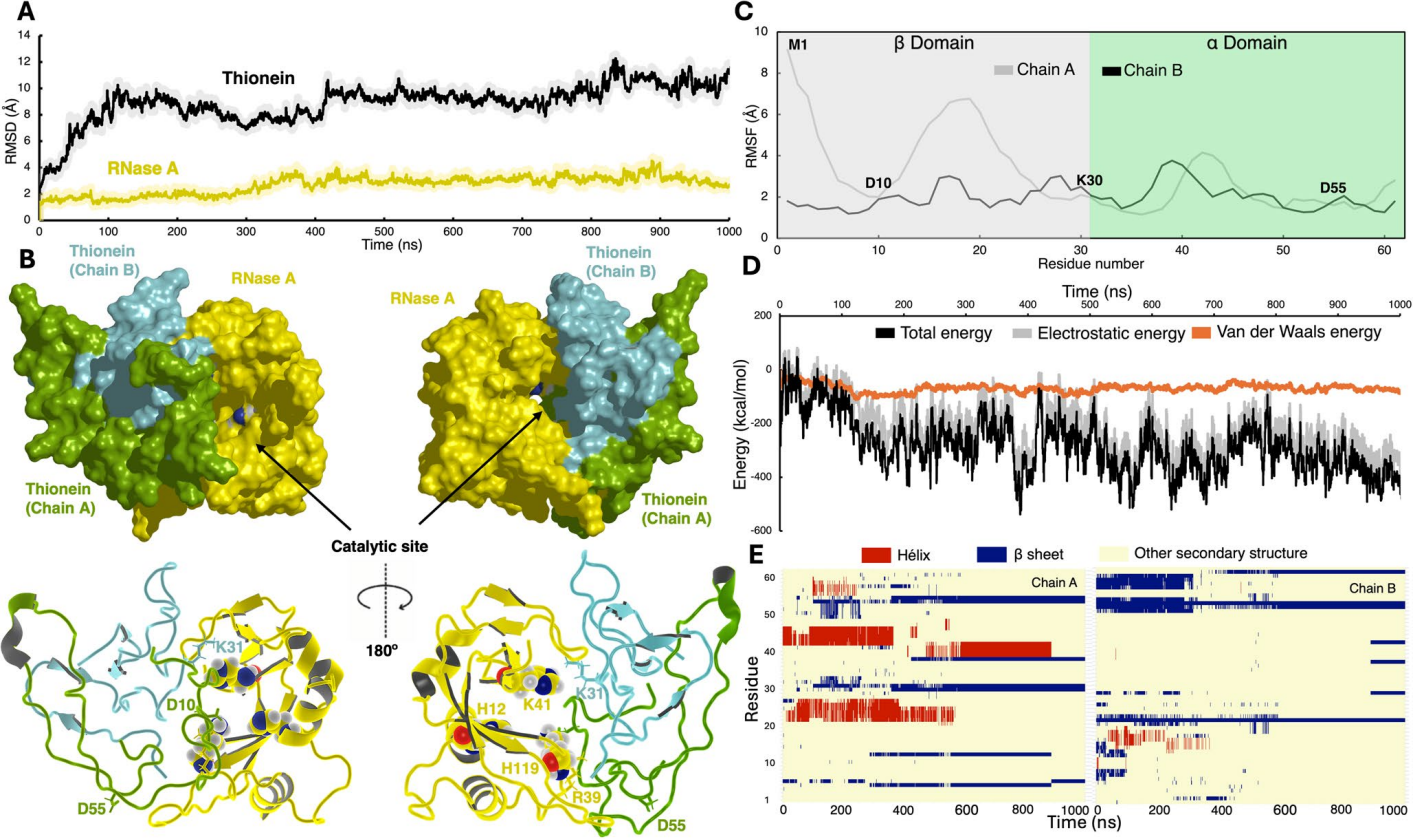
**A** RMSD analysis where T is represented in black and RNase A in yellow. **B** Views of a representative structure of the T-RNase A complex in surface and cartoon representations where the catalytic site is indicated. RNase A is represented in yellow, chain A of T in green and chain B of T in cyan. **C** RMSF analysis of the β and α domains of the chain A and chain B of the dimeric T. Chain A is represented in grey and chain B in black. The position of some important residues that form interactions with RNase A are shown, i.e., Met1, Asp10, Lys30, and Asp55. **D** Interaction NAMD energy analysis between T and RNase A. Total energy is represented in black, electrostatic energy in grey and Van der Waals energy in green. **E** Secondary structure analysis of all the residues of T in the T-RNase A complex, where chain A is shown on the left panel and chain B on the right panel. Helices are represented in red, β sheets in blue and other secondary structures (like coils and turns) in yellow

Based on the RMSD analysis, a stable conformation of the T-RNase A complex was observed. We identified that a structural stabilization is reached at approximately 400 ns for both the Thionein and RNase proteins (see Fig. 6A). As expected and due to its well-known high stability, the RNase protein displayed smaller structural changes (< 4 Å) whereas the homodimeric structure of T explores more diverse conformations (see Fig. 6A). Interestingly, the chain that contributes more to the RMSD values was chain B, which is the one that established a smaller number of contacts with the RNase structure (Figure S8 and S9).

The secondary structure analysis of T in complex with RNase A along the MD simulation showed that the formation of β-strands segments in the domain α of chain B, previously observed in the results from the T homodimer, is maintained during its interaction with RNase (A) However, the analysis also indicates loss of the β-strands in the β domain of chain B (see Fig. 6E). In the case of chain A, the formation of new β-strands on both α and β domains as well as the conservation of previously observed helical segments is observed in the T-RNase A complex. The structure of RNase A presents a kidney shape with a cleft that accommodates the catalytic site residues. The two lobes of the RNase A structure are constituted by loops flanking the β-sheet core that constitutes most of the protein structure. The first RNase A lobe is comprised of two loops—loop1 (L35 to V43) and loop3 (T87 to A96)—that interact mainly with chain A of Thionein. The segment of T’s chain A (55 to 60) that contacts the first lobe shows reduced flexibility as indicated by the calculation of the RMSF (Fig. 6C). The second lobe, mainly formed by loop2 (K61 to N71), contacts Thionein residues from chain (B) The segment of T’s chain B (31 to 37) that interacts with the second lobe also shows reduced flexibility based on its RMSF values (Fig. 6C).

Furthermore, RMSF analysis of other segments of the T homodimer shows that residues present at the protein-protein interface, such as Asp10, Lys30, and Asp55, display smaller fluctuations while the residues in regions that do not establish interactions with RNase A exhibit larger fluctuations (Fig. 6C and S13), highlighting the stability of the interface formed by T and RNase A.

Also, the fluctuation analysis for the different segments of the T homodimer, based on a RMSF calculation, shows that residues present at the protein-protein interface such as Asp10, Lys30, and Asp55, display smaller fluctuations while the residues in regions that do not establish interactions with RNase A exhibits larger fluctuations (Fig. 6C), highlighting the stability of the interface formed by T and RNase A.

To further characterize the stability of the complex formation, we evaluated the protein-protein surface interface by calculating the solvent accessible surface area (SASA). The SASA analyses performed only in the RNase A structure without considering the presence of the T protein, indicates stable values along the simulation (with an average value of 8095.36 ± 1.09 Å^2^), in agreement with the RMSD analysis and its experimentally known high stability. To estimate the interacting protein-protein surface, the solvent-excluded surface (SES), that is, the RNase A surface area that becomes inaccessible to solvent due to the presence of the T protein, was calculated. The SES values remain stable during the simulation time (with an average value of 1426.03 ± 1.04 Å^2^), see Figure S7. When RI binds to ribonucleases, there is a decrease of 2583–3438 Å^2^ in the surface area that is exposed to the surrounding solvent. In comparison, the 1426.03 ± 1.04 Å^2^ value of T is still significant, considering that RI has a greater molecular weight (∼50 kDa), relative to the molecular weight of the T dimer (∼14 kDa) [59, 67, 68]. The SASA analysis in RNase A showed that the protein-protein interface area remains highly stable during the interaction.

To further get insights into the energetic of the protein-protein interaction between RNase A and T, an energy analysis was carried out using the NAMD energy tool [49]. As shown in Fig. 6D, as the simulation proceeds, a decrease (more negative values) in the total interacting energy is observed. We also found that electrostatic energy is the predominant contribution to the stabilization of the protein complex, with a minor contribution from the van der Waals counterpart to the total interacting energy.

Furthermore, since the electrostatic contribution to the total interaction energy was predominant, we analyze the residues directly involved in forming polar interactions, specifically, the formation of salt bridges and H-bonds (see Figure S10, S11 and S12).

The analysis of H-bond formation shows a relatively stable value of the number of H-bonds formed during the MD simulations.

**Fig. 7:**
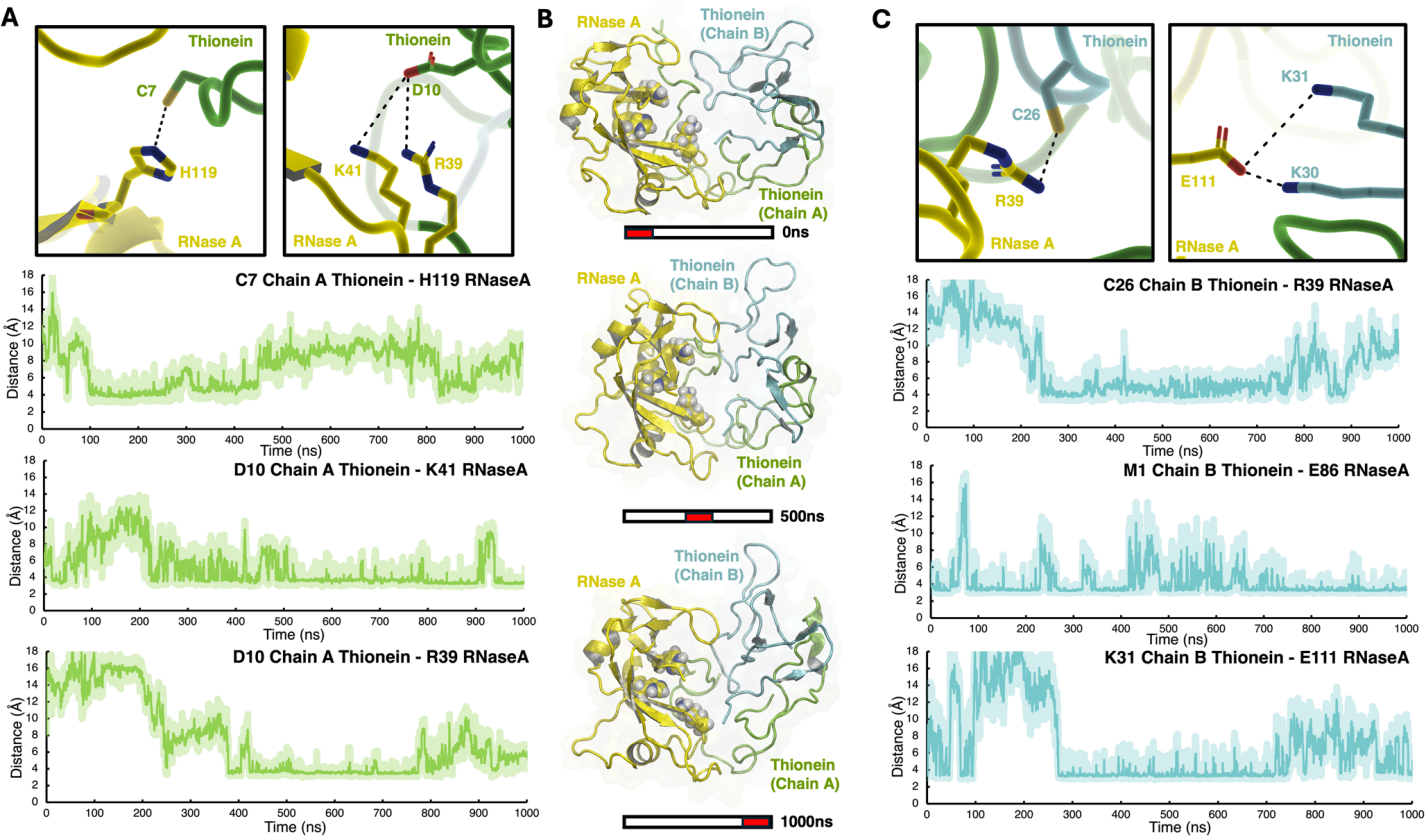
**A** Representative interactions and distances between chain A of T and RNase A. H119, K41, and R39 of RNase A are represented as yellow sticks, C7 and D10 of the chain A of T are represented as green sticks. Time evolution of the distances between C7 (chain A, T)-H119 (RNase A), D10 (chain A, T)- K41 (RNase A) and D10 (chain A, T)-R39 (RNase A) are shown. **B** Representative structures at 0, 500, and 1000 ns at different points along the MD simulation of the T-RNase A complex. RNase A is represented as cartoon in yellow, chain A of T as cartoon in green and chain B of T as cartoon in cyan. **C** Representative interactions and distances between chain B of T and RNase A. R39 and E86 of RNase A are represented as yellow sticks, C26, K30, and K31 of the chain B of T are represented as cyan sticks. Time evolution of the distances between C26 (chain B, T)-R39 (RNase A), M1 (chain B, T)- E86 (RNase A) and K31 (chain B, T)- E111 (RNase A) are shown

In the metal-bound form, MT, all the cysteine residues are unavailable to interact due to their role in coordinating the divalent cations (zinc in our case). In the case of the metal-free form, T, forming a complex with RNase A, we observed that various cysteine residues, including Cys7, Cys15, and Cys26, established H-bond interactions with RNase A (see Fig. 7 and S14).

In the case of the salt bridge formations, the analysis indicated that after 400 ns, the number of charge-charge interactions becomes significant indicating their important contributions to the protein-protein interaction remaining stable for the last 200 ns. When the formation of salt bridges is decomposed in the contribution of chain A and chain B of T, it is evident that the number of charge-charge interactions between chain A and RNase A is larger than those form with chain B, albeit both remain stable in that last segment of the MD trajectory (Figure S12).

Moreover, we identified the formation of stable salt bridges and H-bonds involving residues Arg39 and Lys91, which are residues known to play a central role in the interaction with RI. Along these lines, we also observed an important protein-protein stabilization by the formation of salt bridges between Glu111 from RNase A and Lys30 and Lys31 from chain B of T as well as between Lys41 from RNase A and Asp10 of chain A from T (see Fig. 7). Notably, residues like Lys30 formed intermolecular interactions in the structure of the MT system, which may prevent them from participating in interaction with RNase A in the presence of metals.

The participation of residues from T in the formation of the T-RNase A complex, including Lys30, Cys7, Cys15, and Cys26 all of which formed intermolecular interactions in the metal-bound protein, MT, as well as the structural changes in the structure of the metal-free protein, T, explain why only the T form exhibits interactions with RNase A and the concomitant inhibitory properties observed in our experimental characterization.

## Conclusions

Our in vitro experimental characterization indicates that RNase A interacts specifically with homodimeric metal-free metallothionein (thionein), effectively inhibiting its enzymatic activity. We performed a set of all-atom MD simulations to identify the structural divergence of metal-bound and metal-free MT/T in a physiological context and how these changes correlate with their ability to selectively interact with other proteins. We elucidated representative conformations of T, with the presence of important secondary structural elements which is significantly different from those observed in the MT (e.g., stable β sheets). This information allows us to understand how homodimeric MT/T explores diverse structural conformations that depend on the presence or absence of coordinating metals, allowing it to selectively interact with cytoplasmic proteins such as RNase A. Notably, the presence and function of T under proinflammatory and oxidative stress conditions may constitute a critical component of the cellular defense repertoire, redox regulation, and based on our findings, protein synthesis. This regulation by an alternative mechanism of protein synthesis could be advantageous in various pathophysiological situations characterized by high protein requirements. On the other hand, computational methods like MD simulations provide valuable insights into the molecular mechanisms of protein-protein interactions and offer hypotheses that can be tested using various biophysical and biochemical methods. Understanding the possible interaction of T with other proteins such as other ribonucleases or other proteins that share structural characteristics is interesting for understanding all the signaling pathways in which MT and T participate. While the integration of in vitro assays and computational modeling provides compelling evidence for the inhibitory capacity of apo-metallothionein against the enzymatic activity of RNase A, several limitations justify further study. Specifically, the biological relevance of this inhibition under cell-based and in vivo conditions remains to be elucidated. Furthermore, the potential for RNase A to form oligomers and the subsequent impact of such assemblies on its interaction with thionein must be addressed. Investigating these factors is essential to clarify the physiological implications of RNase A inhibition, particularly during the acute-phase response where *de novo* protein synthesis is dynamically regulated.

## Supplementary Information

Below is the link to the electronic supplementary material.

Supplementary Material 3 (DOCX 3,998 KB)

## Data Availability

Data will be shared upon a reasonable request.
