# Supplementary material for: RNase A is inhibited by the cysteine-rich protein thionein but not by the metal-containing form metallothionein
Source: Mol Cell Biochem. 2026 Mar 13;481(5):1989–2003. doi: 10.1007/s11010-026-05515-z (PMC13179892; doi:10.1007/s11010-026-05515-z)
Supplement: Supplementary file 1 — Supplementary Material 3 (DOCX 3,998 KB) [file 11010_2026_5515_MOESM1_ESM.docx]

**Supplemental Information**

**RNase A is Inhibited by the Cysteine-Rich Protein Thionein but not by the Metal-Containing Form Metallothionein.**

Francisco Trujillo-González^1^, Ian Asaf Muñoz-Granados^1^, Brenda L. Sanchez-Gaytan^2^, Eduardo Brambila ^1,*^, Jose Manuel Perez-Aguilar^1,*^.

**Figure S1.** **Sephadex G-75 Chromatography.**

**Figure S2.** **Ribonuclease inhibition by the Apo-thionein-1 (MT-1).**

**Figure S3.** **RNase activity restoration after Zn addition.**

**Figure S4.** **Structural alignment of the 4MT2 X-ray structure of MT2 and a representative structure of the MT2 dimer from the MD simulation.**

**Figure S5.** **Representative structures of the best poses obtained by the consensus protein-protein docking scheme.**

**Figure S6** **Structural alignment of the RNase 1-RI and RNaseA-T complexes.**

**Figure S7.** **SASA and SES analysis of RNAse A**.

**Figure S8. Analysis of the number contacts at 5 Å of the chain A of T and RNaseA.**

**Figure S9. Analysis of the number contacts at 5 Å of the chain B of T and RNaseA.**

**Figure S10. Analysis of the number of H bonds between T and RNaseA.**

**Figure S11. Analysis of the number of salt bridges between T and RNaseA.**

**Figure S12. Analysis of the number of salt bridges between T (Chain A and Chain B) and RNaseA.**

**Figure S13. Distance between D55 of chain A of T and K91 of RNaseA.**

**Figure S14. Distance between C15 of chain A of T and K7 of RNaseA.**


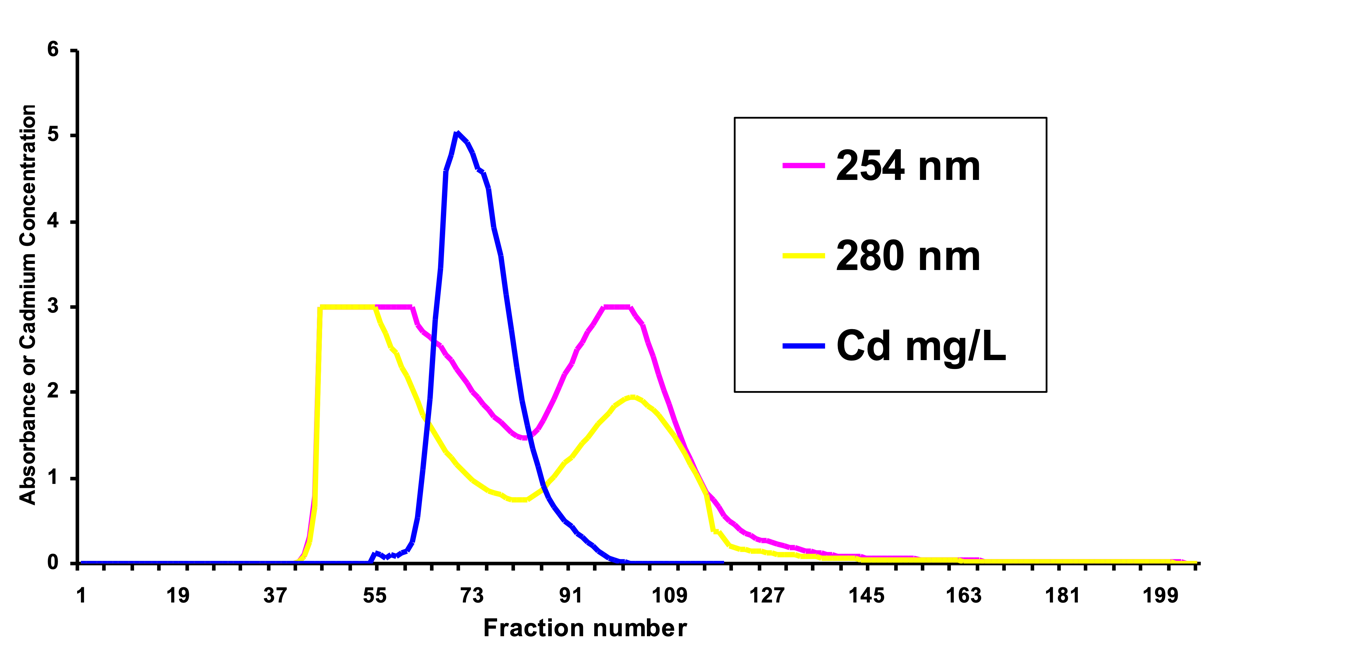
**Figure S1.** **Sephadex G-75 Chromatography**. MT was isolated using a 10mM Tris-HCl buffer. The flow rate was 0.6 mL/min and 6.0 mL fractions were collected. The presence of MT in fractions was monitored by the UV absorption at 254 nm and MT-Cd content by atomic absorption.


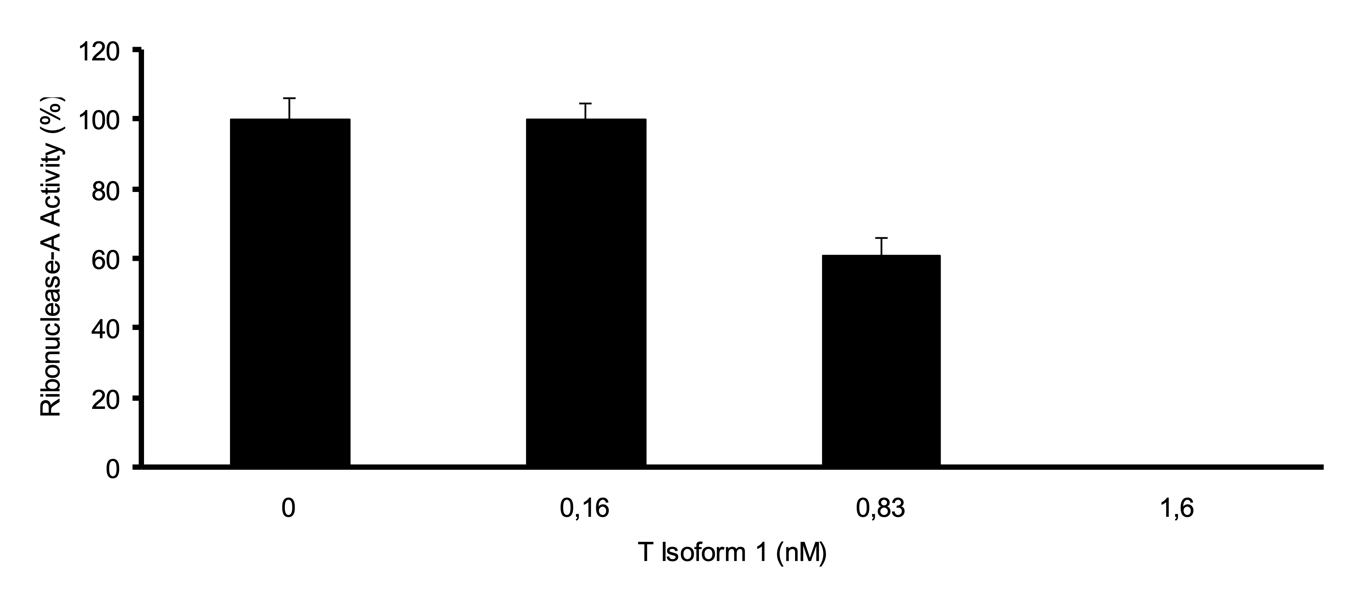


**Figure S2.** **Ribonuclease inhibition by the Apo-thionein-1 (MT-1),** RNase activity was determined by measurement of 2´, 3´-cCMP in Tris/acetate buffer pH 6.5 at 25 ºC hydrolysis after addition of varying amounts of MT to the solution. The product 3´CMP was measured at 286 nm. Apo MT-1 shows the same inhibitory effect on RNase activity, such as total Thionein, T. Data represent means (N=3).


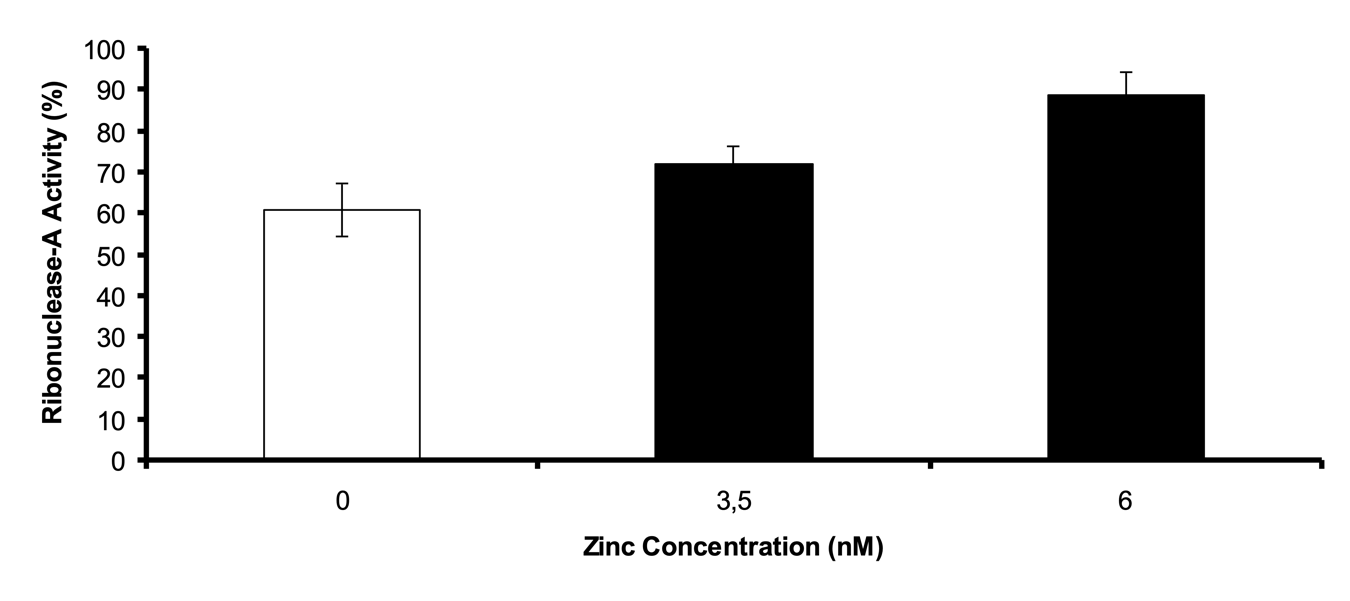


**Figure S3.** **RNase activity restoration after Zn addition.** Zn addition to the solution containing partially inactivated RNase (61% activity) restore enzymatic activity. Maximum RNase reactivation (89%) was obtained after the addition of 6 nM of Zn to the solution. Data represent means (N = 3).


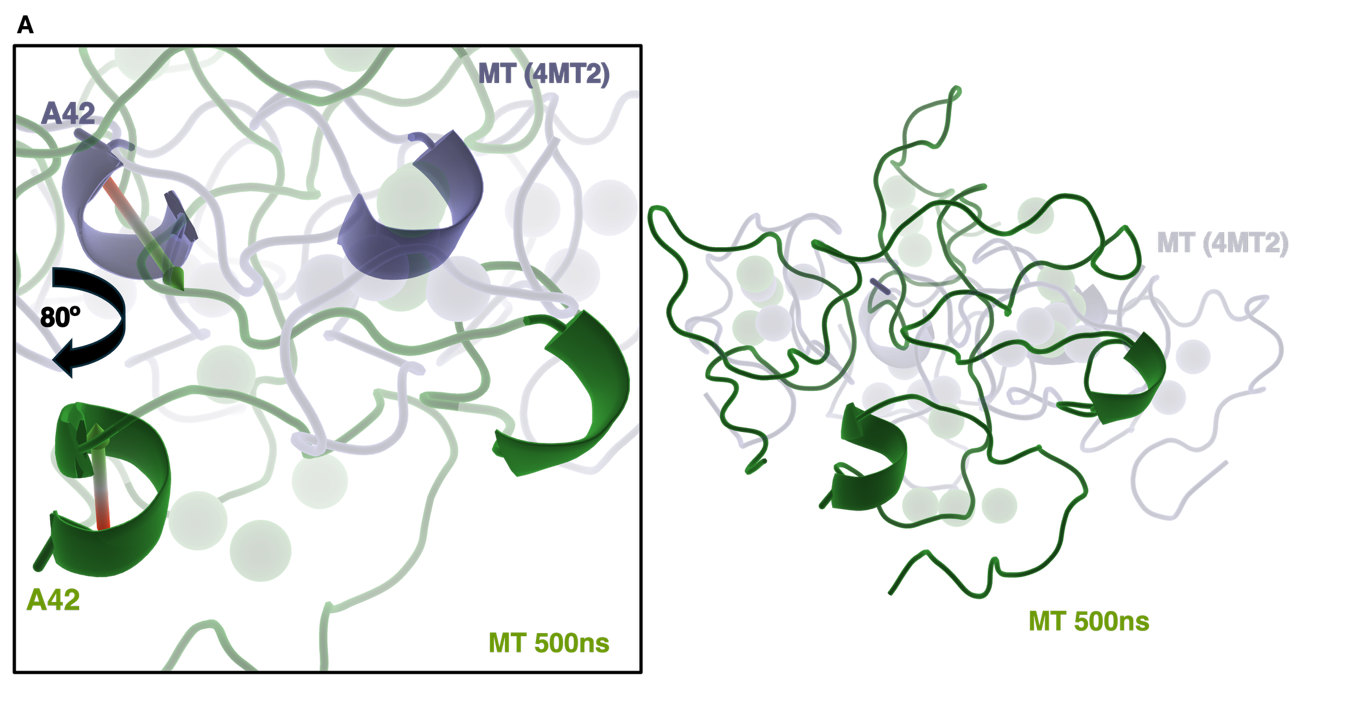
**Figure S4.** **Structural alignment of the 4MT2 X-ray structure of MT2 and a representative structure of the MT2 dimer from the MD simulation.** Superposition of the structures of the MT homodimer (PDB accession code 4MT2) and a representative structure of the MT homodimer at 500 ns of the MD simulation. 4MT2 is colored purple and the structure of MT from the MD simulation (at 500 ns) is colored green. On the left, the inset panel shows a representative residue (Ala42) from the short alpha-helical segment of the structure of MT2 (around residues 42 to 45) and its rotation in the MD simulation. The structure of both monomers remains stable, with a rotation of approximately 80º of one chain with respect to the other.


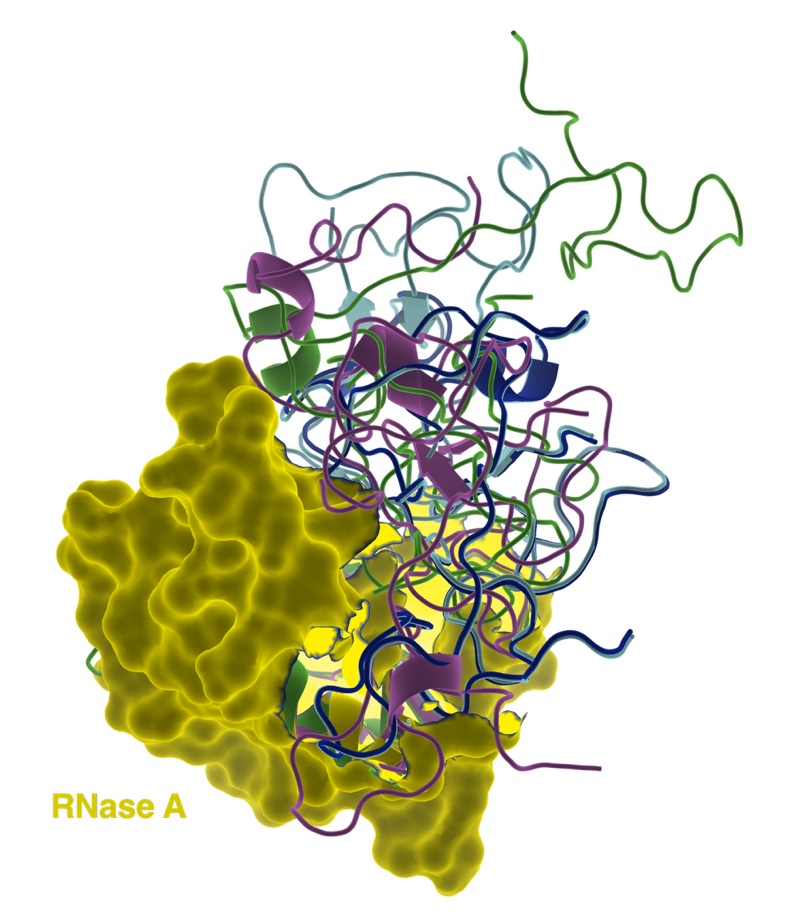


**Figure S5.** **Representative structures of the best poses obtained by the consensus protein-protein docking scheme.** Superposition of the docking poses of the structures of the T homodimer and RNase A. RNase A is represented as a surface and colored yellow and all the poses obtained from T are represented as a cartoon oriented towards RNase A alignment.

**Figure S6** **Structural alignment of the RNase 1-RI and RNaseA-T complexes.** Superposition of the structures of the RNase 1-RI complex (PDB accession code 1Z7X) and a representative structure of the T-RNase A complex from the microsecond-long MD simulations. RNase A is colored yellow, RNase 1 is colored purple, RI is colored gray and Thionein is colored green and cyan. C and D panels show equivalent residues that mediate the interaction of the RNases with RI and Thionein, including charge-charge interactions with Arg39 and Lys91 (Arg91 in RNase 1 is replaced by a Lys91 in RNase A, maintaining its positively charged sidechain). In the case of the experimentally-solved RNase 1-RI structure, R39 (RNase 1) forms a salt-bridge with E401(RI) and R91 (RNase 1) forms a salt-bridge with E287(RI); both interactions contribute to the stability of the complex (indicated by green dashed lines). From our results, we observed that Thionein also establishes equivalent charge-charge interactions with RNase A – R39(RNase A) interacts with D10 while K91(RNase A) interacts with D55 (indicated by black dashed lines).


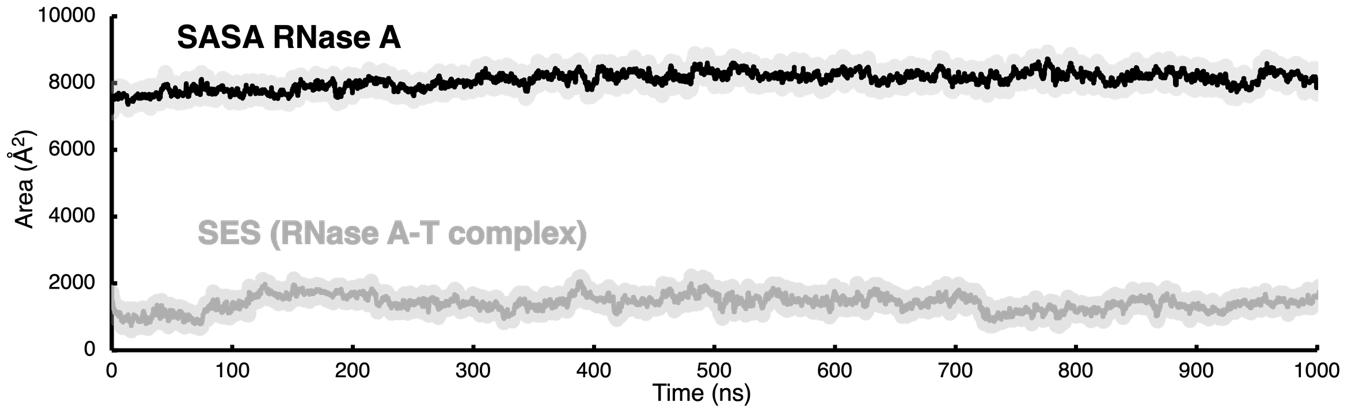


**Figure S7.** **SASA and SES analysis of RNAse A**. The time evolution of the RNAse A solvent accessible surface (SASA) and the solvent-excluded surface due to the interaction with the T dimer (SES) using the radio of 1.4 Å^2^ (that of the water molecule) is shown. The SASA displays stable values with an average value of 8095.36 ± 1.09 Å^2^. In the presence of T, the RNAse A solvent-excluded surface (SES), which indicates the surface area that becomes inaccessible to the solvent due to the presence of T, exhibits stable values with an average value of 1426.03 ± 1.04 Å^2^. Both parameters indicate stable protein-protein interactions in the RNaseA-T complex.


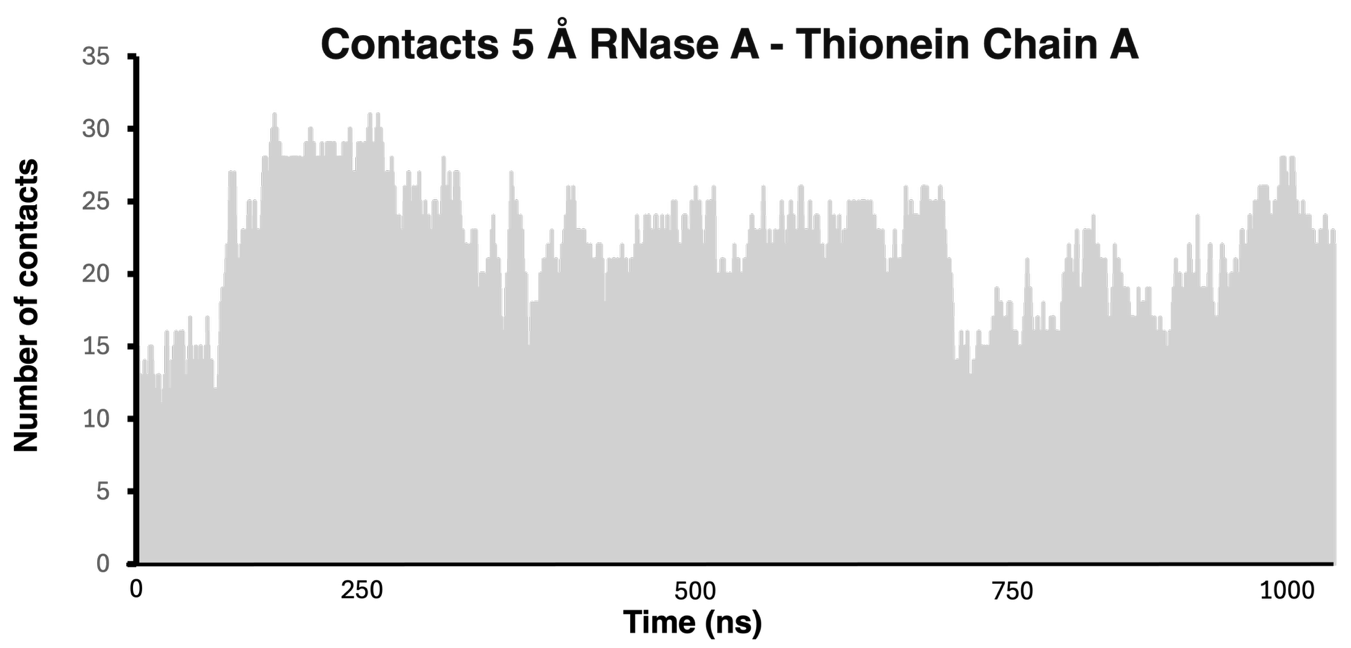


**Figure S8. Analysis of the number contacts at 5 Å of the chain A of T and RNaseA.** Time evolution plot of the number of residues of the chain A of T that are within 5 Å from RNaseA.


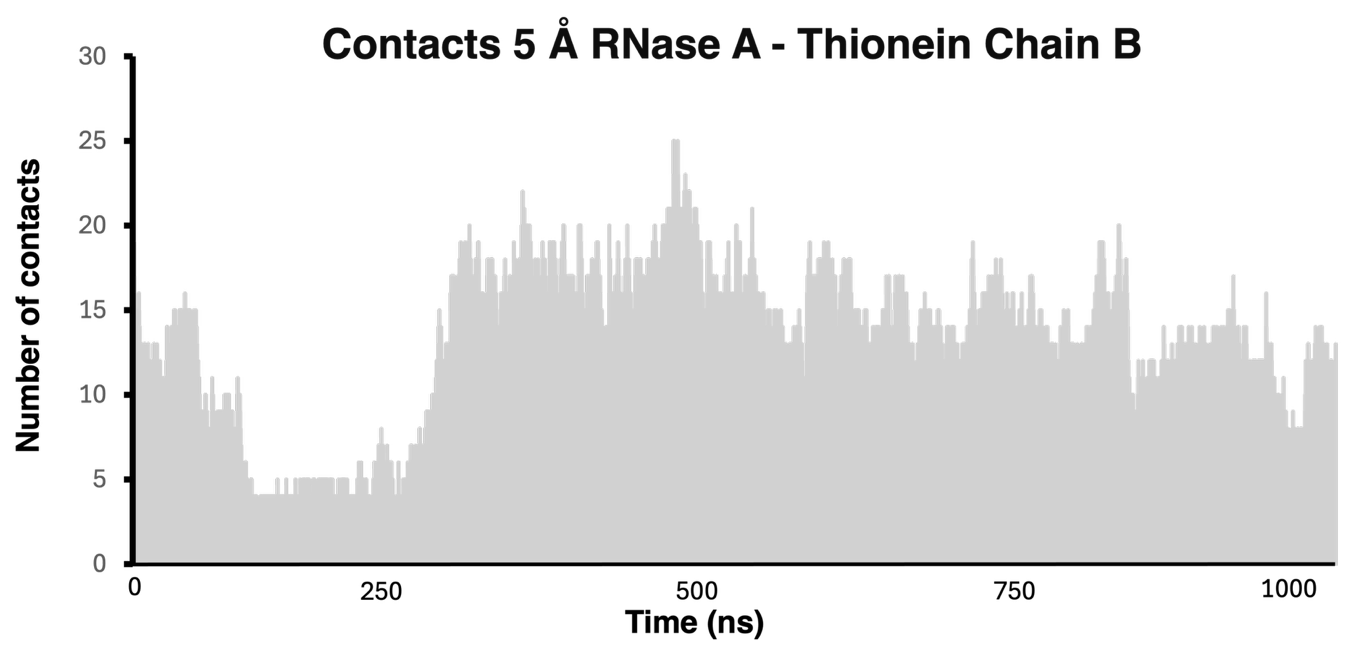


**Figure S9. Analysis of the number contacts at 5 Å of the chain B of T and RNaseA.** Time evolution plot of the number of residues of the chain B of T that are within 5 Å from RNaseA.


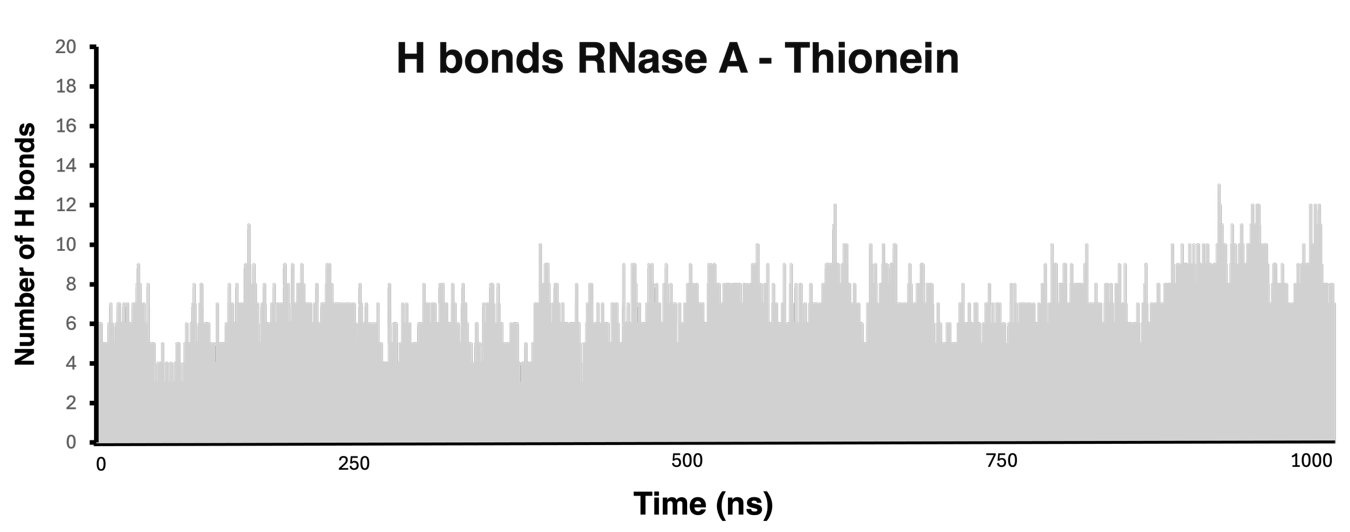


**Figure S10. Analysis of the number of H bonds between T and RNaseA.** Time evolution plot of the number of H bonds between dimeric T and RNaseA. The trend in the number of H bonds suggests stable protein-protein interactions.


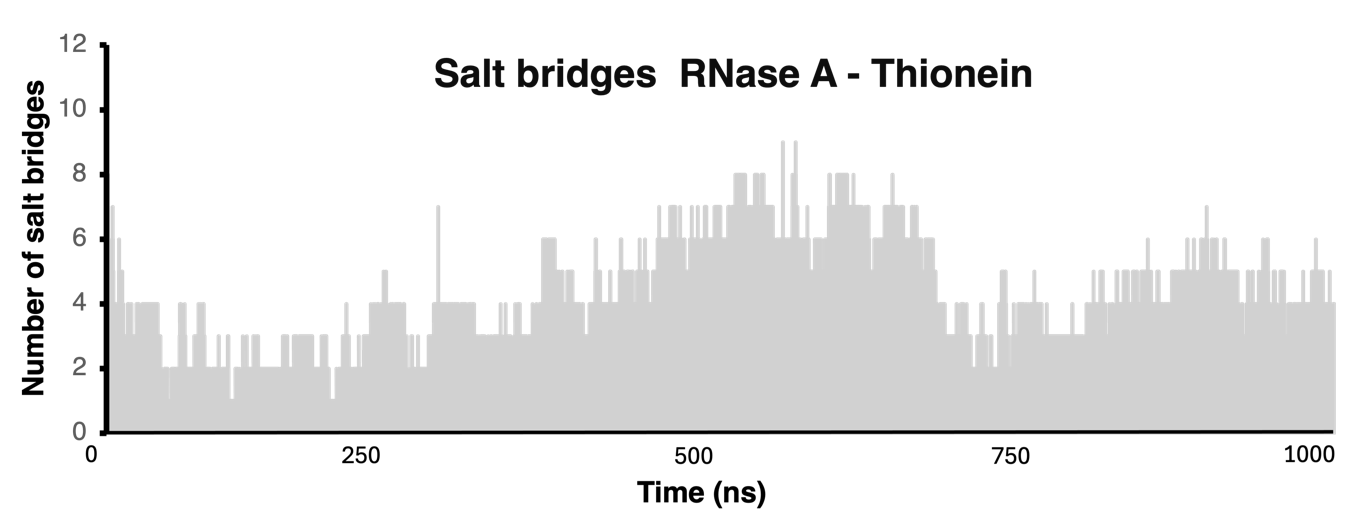


**Figure S11. Analysis of the number of salt bridges between T and RNaseA.** Time evolution plot of the number of salt bridges between dimeric T and RNaseA. The value for the last 200 ns seems stable.


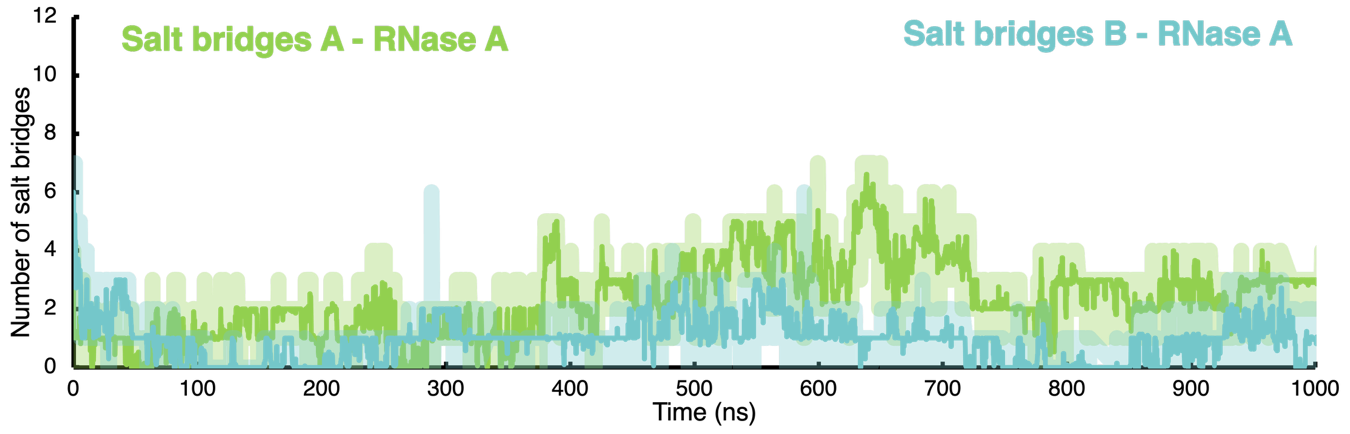


**Figure S12. Analysis of the number of salt bridges between T (Chain A and Chain B) and RNaseA.** Time evolution plot of the number of salt bridges between Chain A and Chain B of T and RNaseA.


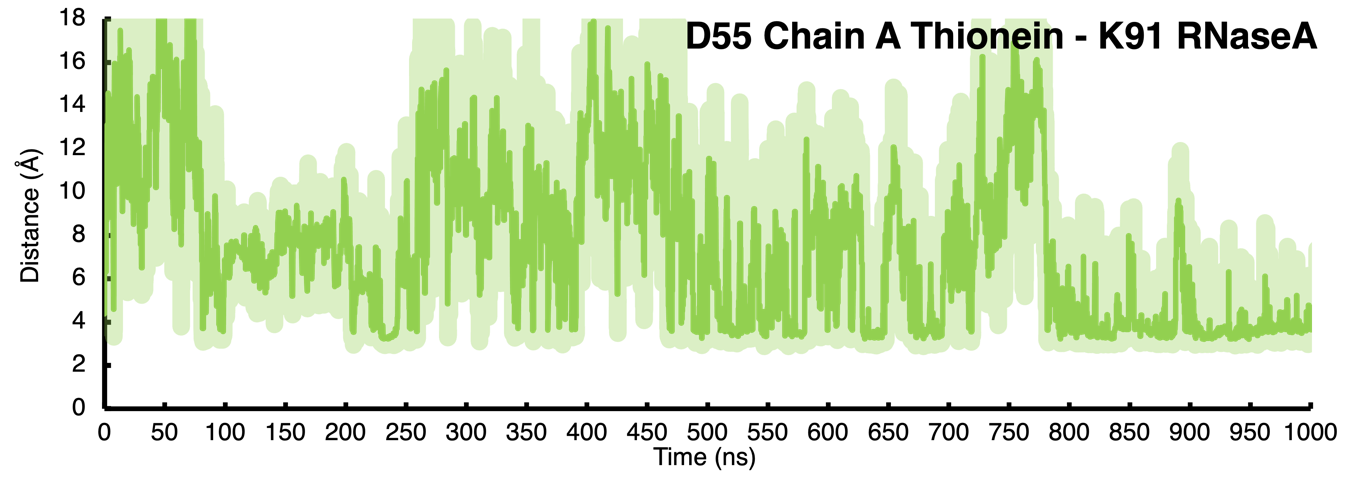


**Figure S13. Distance between D55 of chain A of T and K91 of RNaseA.** Time evolution of the distance between the positively-charged K91(RNase A) and the negatively-charged D55(chain A of T). The interaction starts at early stages of the simulation and stabilizes during the last 200 ns.


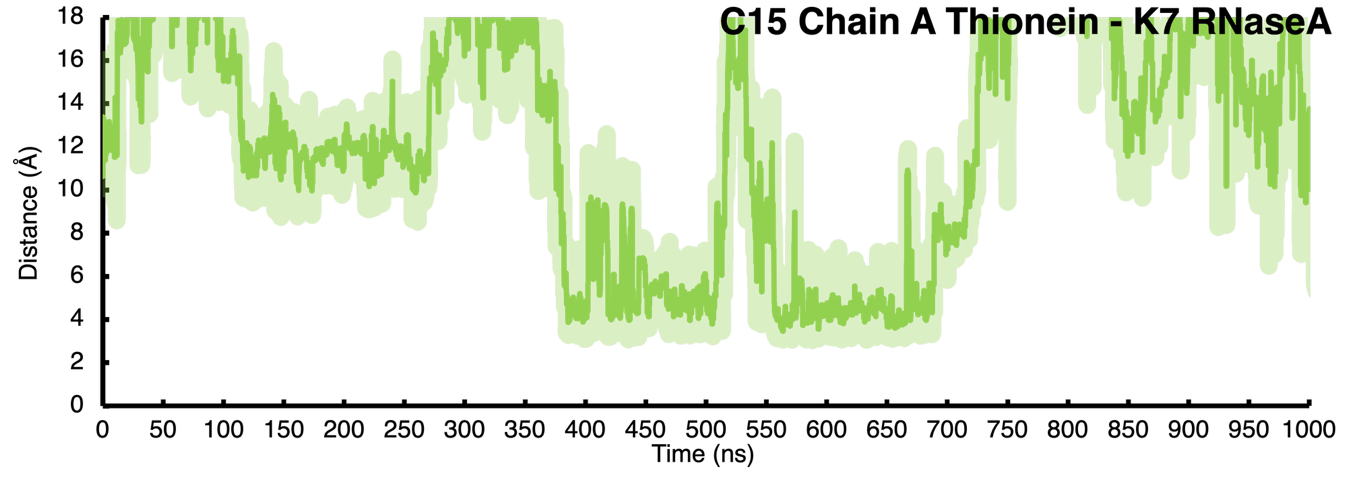


**Figure S14. Distance between C15 of chain A of T and K7 of RNaseA.** Time evolution of the distance between the positively-charged K7(RNase A) and C15(chain A of T). The interaction is maintained between 400 and 700 ns.
